# Stereo Vision-Based High Dynamic Range Imaging Using Differently-Exposed Image Pair

**DOI:** 10.3390/s17071473

**Published:** 2017-06-22

**Authors:** Won-Jae Park, Seo-Won Ji, Seok-Jae Kang, Seung-Won Jung, Sung-Jea Ko

**Affiliations:** 1 School of Electrical Engineering, Korea University, 145 Anam-ro, Seongbuk-gu, Seoul 136-701, Korea; wjpark@dali.korea.ac.kr (W.-J.P.); swji@dali.korea.ac.kr (S.-W.J.); 2 Samsung Electronics Co. Ltd., 1, Samsungjeonja-ro, Hwaseong-si 445-330, Gyeonggi-do, Korea; seokjae.kang@samsung.com; 3 Department of Multimedia Engineering, Dongguk University, Pildong-ro 1gil 30, Jung-gu, Seoul 100-715; Korea; swjung83@dongguk.edu

**Keywords:** high dynamic range imaging, high dynamic range reconstruction, stereo matching, stereo vision system, hole-filling

## Abstract

In this paper, a high dynamic range (HDR) imaging method based on the stereo vision system is presented. The proposed method uses differently exposed low dynamic range (LDR) images captured from a stereo camera. The stereo LDR images are first converted to initial stereo HDR images using the inverse camera response function estimated from the LDR images. However, due to the limited dynamic range of the stereo LDR camera, the radiance values in under/over-exposed regions of the initial main-view (MV) HDR image can be lost. To restore these radiance values, the proposed stereo matching and hole-filling algorithms are applied to the stereo HDR images. Specifically, the auxiliary-view (AV) HDR image is warped by using the estimated disparity between initial the stereo HDR images and then effective hole-filling is applied to the warped AV HDR image. To reconstruct the final MV HDR, the warped and hole-filled AV HDR image is fused with the initial MV HDR image using the weight map. The experimental results demonstrate objectively and subjectively that the proposed stereo HDR imaging method provides better performance compared to the conventional method.

## 1. Introduction

Most commercial charge coupled device (CCD) or complementary metal-oxide semiconductor (CMOS) sensors deliver a limited dynamic range (DR) which is usually several orders of magnitude lower than that of a real scene. To overcome such limitation of image sensors, many researchers have developed various DR extension methods, which are also called high dynamic range (HDR) imaging. While some approaches enhance the DR by using particular sensors [1,2,3], other HDR imaging methods use image processing techniques to generate a high-quality HDR image from low dynamic range (LDR) images captured by low-cost cameras. These HDR imaging methods use multiple LDR images of the same scene captured under different exposures and fuse them into the HDR image [4,5,6,7]. Thus, the resultant HDR image has a wide DR similar to a real scene. However, since the fusion process assumes that the scene is completely static, the faint appearance of objects, called ghosting artifact, is often observed in the final HDR image especially when the scene contains moving objects. Jacob and coworkers’ method [8] attempts to reduce the ghosting artifact by explicitly detecting and removing the moving objects when combining multi-exposed images, but moving object detection itself is challenging especially for multi-exposed images. Other ghost removal algorithms [9,10] use the PatchMatch-based global optimization to obtain the artifact-free HDR image, but the algorithms generally require high computational cost. Although the PatchMatch-based global optimization can be accelerated by Lu and coworkers’ method [11], which uses the super-pixel as a basic unit for the PatchMatch, super-pixel segmentation itself is challenging for the images captured under the limited dynamic range. The ghosting artifact can also be removed by using special sensors that support spatially varying pixel exposures [12]. However, such sensors not only increase the cost of the cameras but also reduce the resolution of the resultant HDR image.

In an attempt to deal with the ghosting artifact using general sensors, several researchers have focused on reconstructing an HDR image from a stereo image pair captured with different exposures, called stereo HDR imaging [13,14,15,16]. According to the literature survey [17], these methods [13,14,15,16] correspond to the single source method because a single input image is used from each camera. One method [13] uses the left HDR image and the right LDR image to generate the right HDR image, and the others [14,15,16] use a stereoscopic LDR image pair as an input. Since the input LDR image pair is captured together with slightly different exposures, the ghosting artifact can be largely alleviated in the resultant HDR image. However, the input LDR images of the stereo HDR imaging system have different viewpoints with each other. Thus, in stereo HDR imaging, two challenging issues have to be addressed: First, high-performance disparity estimation is required to align the AV with the MV. Second, an effective hole-filling method has to be employed to restore the radiance values of the hole regions in the HDR image.

The proposed HDR imaging method adopts a basic framework of the conventional stereo HDR imaging methods [14,15,16] which consist of image rectification, radiance space conversion, disparity estimation, image warping, and image fusion. In the proposed method, we reconstruct the final HDR image with considering the aforementioned two challenging issues. First, the human visual system (HVS)-based cost computation and segmentation-based cost aggregation method are proposed to obtain a more precise disparity map. Second, the effective hole-filling method is proposed to compensate for the incomplete region in the warped AV HDR image.

The remainder of this paper is organized as follows. The proposed stereo HDR imaging is described in Section 2. In Section 3, experimental results are presented and discussed. Finally, this paper is concluded in Section 4.

## 2. Proposed Stereo HDR Imaging Method 

### 2.1. Overall Framework

**Figure 1:**
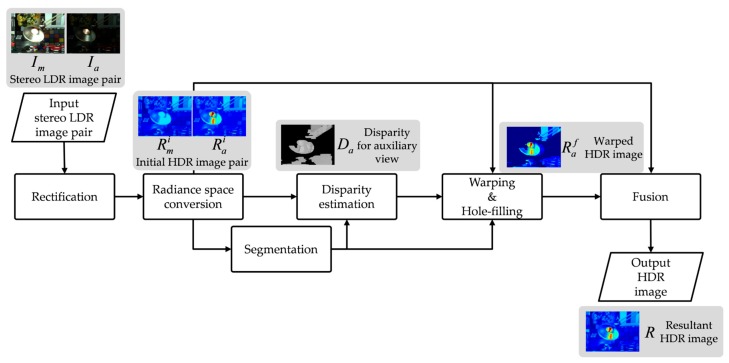
A framework of the proposed stereo high dynamic range (HDR) imaging.

An overall framework for the proposed stereo HDR imaging method is shown in Figure 1. The framework of the proposed method is similar to the flow of conventional stereo HDR imaging methods [14,15,16]. First, a stereo image pair is transformed into a common image plane using the well-known rectification method [18] prior to the stereo matching process. A stereo LDR image pair of the MV image *I_m_* and the AV image *I_a_* is assumed to be horizontally aligned by rectification. Next, both *I_m_* and *I_a_* are used to obtain the inverse camera response function (ICRF) [19]. After obtaining the initial HDR images by the radiance space conversion, the resultant HDR images of the proposed stereo HDR imaging method are obtained by three major sub-processes: (1) disparity estimation; (2) warping and hole-filling; and (3) fusion with the warped and hole-filled AV HDR image. The segmentation results of the initial HDR images are also used as supplementary information in the first two sub-processes, as shown in Figure 1.

**Figure 2:**
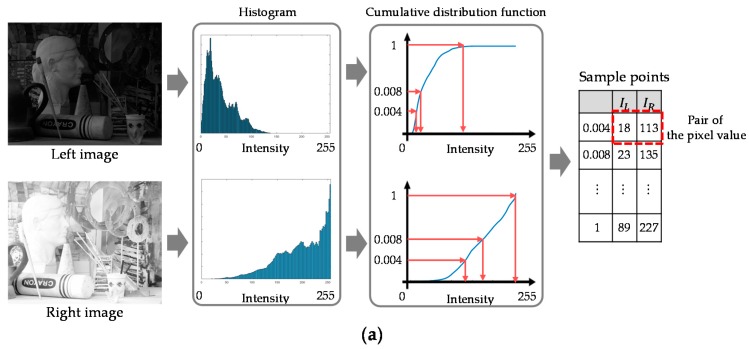

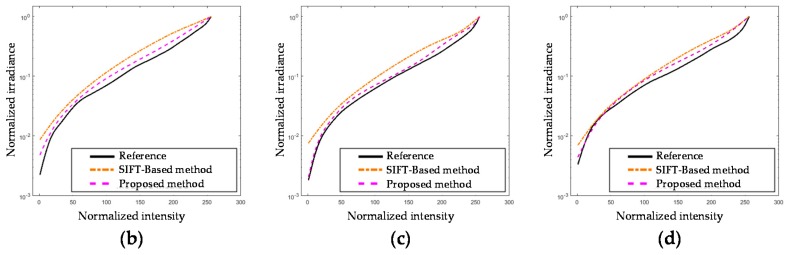
(**a**) An example of the proposed method; and the obtained inverse camera response functions (ICRFs) of: (**b**) the red channel; (**c**) the green channel; and (**d**) the blue channel.

However, since the camera response function (CRF) estimation method [19] is presented for the single-view images with different exposure times, called the bracketed images, the method should be modified for the stereo HDR imaging system. To apply the CRF estimation method [19] to the stereo HDR imaging system, it is mandatory to obtain the pixel correspondences between the stereo images, which are called sample points [14]. The conventional stereo HDR imaging method [14] employs the SIFT descriptor to find the sample points. However, the conventional method [14] does not guarantee the sample points for the full intensity range due to using the SIFT descriptor. To obtain enough sample points for the entire intensity range, the proposed HDR imaging method utilizes the cumulative distribution functions (CDFs) of the stereo images. The proposed method assumes that the pixel values with the same probability at two different CDFs of the stereo images have the similar irradiance value. Thus, given the two CDFs, the sample points are collected by selecting the pairs of pixel values that correspond to the same probability. For example, the intensity value 18 of the left image is matched with the intensity value 113 of the right image, as shown in Figure 2a. All the collected sample points are then used to estimate the ICRF [19]. Figure 2b–d shows the resulting ICRF curves of the conventional method [14], the proposed method, and the reference method [19]. The ICRF curves of the conventional and the proposed method are obtained by using the stereo images with two different exposure times (short and long). The reference ICRF [19] is generated by using the single-view images with the three different exposure times (short, normal, and long). It can be seen that the ICRFs of the proposed ICRF method for the stereo HDR imaging system are more accurate than the conventional ones [14].

**Figure 3:**
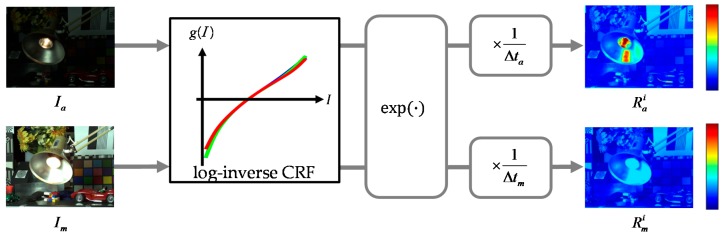
Radiance space conversion.

Figure 3 shows a radiance space conversion process. The rectified LDR images are first converted to the initial HDR images, $R_{m}^{i}$ and $R_{a}^{i}$, by using the proposed ICRF method. These initial HDR images are then used for the proposed stereo HDR imaging system.

Similar to the conventional algorithms [14,15,16], the left-view image and the right-view image are set as the MV image and the AV image, respectively. Although the initial HDR images are obtained using the estimated ICRF, the DR of the initial HDR images is inherently limited by the input LDR images, which is especially noticeable in the under/over-exposed regions. Thus, to expand the DR of the MV image $R_{m}^{i}$, the under/over-exposed regions are detected and restored from the corresponding regions in the initial AV HDR image $R_{a}^{i}$.

**Figure 4:**
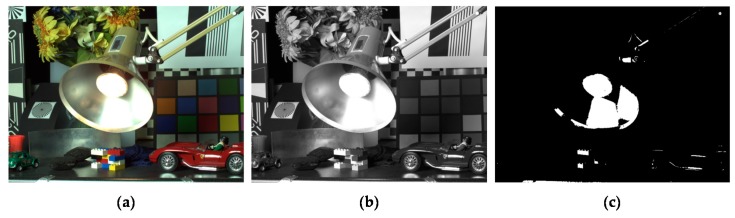
(**a**) Input MV LDR image, *I_m_*; (**b**) luminance channel of *I_m_* and *L_m_*; and (**c**) under/over-exposed region map, *S*.

The under/over-exposed regions are detected from *I_m_*, as follows:

(1) $$S(p)=\{\begin{array}{cc} 1, & \mathrm{if}\;0\;<L_{m}(p)<\tau_{u}\;\mathrm{or}\;\tau_{o}<L_{m}(p)<255\\ 0, & \mathrm{otherwise} \end{array},$$

where *S*(·) represents a binary under/over-exposed region map (1: under/over-exposed, 0: otherwise). *τ_u_* and *τ_o_* are the thresholds to determine the under/over-exposed regions. **p** denotes the 2D pixel coordinates and *L_m_* denotes the luminance value of *I_m_*. Figure 4c shows the detected under/over-exposed region map.

The corresponding under/over-exposed regions need be detected from the AV image in order to restore the radiance values of the under/over-exposed regions. For this purpose, per-pixel disparity of the AV image, *D_a_*, is required. We estimate the disparity by combining the HVS-based cost measure and the segmentation-based aggregation method. The proposed disparity estimation method is described in detail in Section 2.2.

**Figure 5:**
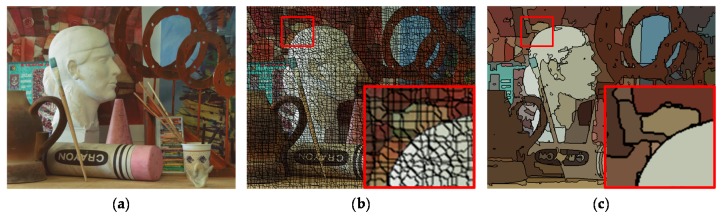
An example of the segmentation process: (**a**) input image; (**b**) resultant image of the super-pixel lattices [20]; and (**c**) resultant image of the graph-based region merging [21].

Unlike the conventional stereo HDR imaging methods [14,15,16], the segmented images are used in the proposed stereo HDR imaging method. Prior to disparity estimation, the segmented images are obtained using the two-step segmentation. To speed up the segmentation process, the initial HDR images are over-segmented into super-pixels using the super-pixel lattices (SL) method [20], as shown in Figure 5b. Since the SL method segments the image into a regular grid of super-pixels, it can be easily adapted to the graph-based algorithm. The obtained super-pixels are processed as the input pixels of the graph-based region merging (GRM) method [21]. The resultant super-pixels of the SL method are grouped into segments using the GRM to generate the segmented MV image *G_m_* and AV image *G_a_*, as shown in Figure 5c. These segmentation results are used for the disparity estimation and the hole-filling.

After obtaining the segmentation results and the AV disparity *D_a_*, image warping is performed to align the AV HDR image with the MV HDR image. Then, the warped AV HDR image $R_{a}^{w}$ is compensated by the hole-filling process. The proposed hole-filling method and the fusion process to reconstruct the final HDR image are presented in detail in Section 2.3.

### 2.2. Disparity Estimation

**Figure 6:**
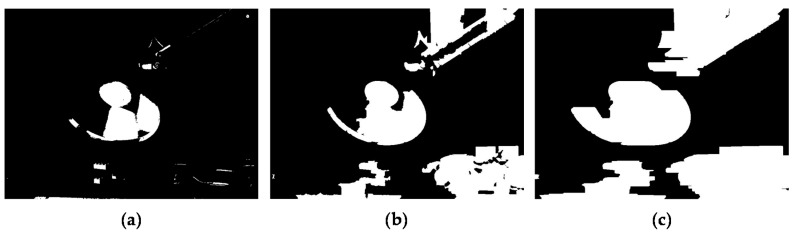
(**a**) Under/over-exposed region map, *S*; (**b**) a set of all the segments that contain the under/over-exposed pixels; and (**c**) interest regions.

Prior to the estimation of the AV disparity *D_a_*, the regions of interest in the AV image are first detected from Equation (1). To do this, the segments in *G_m_* that include the pixels in the under/over-exposed regions are selected. The pixels in these segments are determined as interest pixels in the AV image as shown in Figure 6b. Then, each interest pixel is expanded to the horizontal direction with the maximum disparity, *d*_max_, as shown in Figure 6c for the estimation of the AV disparity. The proposed method only estimates the disparity of the interest regions to reduce the complexity.

Since it is shown that stereo matching can better perform with the HDR images than the LDR images [22], we also perform stereo matching using the initial HDR images, $R_{m}^{i}$ and $R_{a}^{i}$. To compute the initial matching cost *C*(**p**,*d*) at pixel **p** for the disparity value *d* ∈ [0, *d*_max_], the census transform [23] is used. The performance of the census transform-based cost computation is reported to be superior to other matching cost computation methods for the images with radiometric variations [24]. In the census transform, a bit string is defined by a center pixel **p** and pixel **q** ∈ *N*(**p**), where *N*(**p**) is a set of pixels around **p**. Each bit is set to 0 if the intensity of the corresponding pixel **q** is lower than that of the pixel **p** and set to 1 otherwise. The matching cost between the pixel **p** in the AV image and a candidate pixel **p’** in the MV image is measured by computing the Hamming distance between corresponding bit strings.

**Figure 7:**
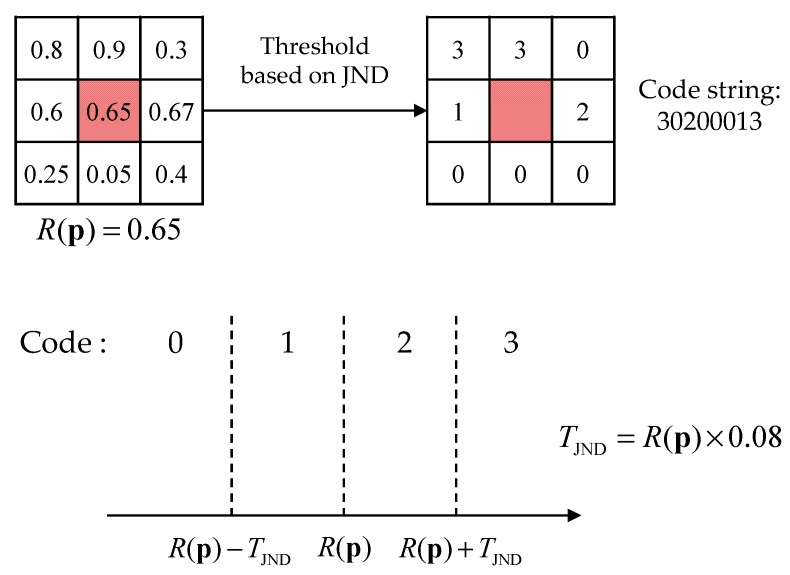
The process of human visual system (HVS)-based cost computation in a 3 × 3 local region.

In the proposed disparity estimation process, each bit of the census transform is converted into four-valued code by using a threshold, as shown in Figure 7. Moreover, the threshold for the four-valued census transform is obtained by a perceptual threshold, which is called just noticeable difference (JND). The resultant eight codes are concatenated as follows:

(2) $$s(p)=\overset{}{\underset{q\in N(p)}{\left|\right|}}s_{c}(p,q),$$

where

(3) $$s_{c}(p,q)=\{\begin{array}{cc} \begin{array}{c} 0,\\ 1,\\ 2,\\ 3, \end{array} & \begin{array}{l} \mathrm{if}\;R(q)<R(p)-T_{\mathrm{JND}}(p)\\ \mathrm{if}\;R(p)-T_{\mathrm{JND}}(p)\leq R(q)<R(p)\\ \mathrm{if}\;R(p)\leq R(q)\leq R(p)+T_{\mathrm{JND}}(p)\\ \mathrm{if}\;R(q)<R(p)-T_{\mathrm{JND}}(p)\; \end{array} \end{array},$$

where

(4) $$T_{\mathrm{JND}}(p)=R(p)\times 0.08,$$

where || denotes concatenation, *s_c_*(**p**,**q**) is the four-valued code of **p** and **q**. *s*(**p**) is the eight code string of **p**, *R*(**p**) is radiance value of p, and *T_JND_*(**p**) is the threshold value for p by the JND. As a result, the number of coincident codes between the code strings of radiance becomes the matching cost.

Given the initial matching cost *C*(**p**,*d*) at the pixel **p** for disparity value *d*, cost aggregation is performed using the adaptive support-weight approach [25]. The adaptive support-weights of the pixels in a given support window using the color similarity and geometric distance are used to increase the reliability of the disparity map *D_a_* [25], as follows:

(5) $$w_{a}(p,q)=exp\left(-\left(\frac{{\left\|I(p)-I(q)\right\|}_{2}}{\varepsilon_{I}}+\frac{{\left\|p-q\right\|}_{2}}{\varepsilon_{S}}\right)\right),$$

where *w_a_* is the adaptive support-weight function and ‖·‖_2_ denotes the L-2 norm. **p** and **q** denote a central pixel position and a neighboring pixel position of **p**, respectively, and *I*(**p**) represents a color components of **p**. *ε_I_* and *ε_S_* denote predefined parameters. Since the proposed method already groups the pixels into segments, it can be assumed that the pixels in the same segment have similar disparity values [26]. Thus, the weight function *w_s_* is defined as follows:

(6) $$w_{s}(p,q)=\exp\left(-\frac{{\left\|p-q\right\|}_{2}}{\sigma^{2}}\right),$$

where *σ*^2^ represents the variance of *w_s_*(·). Then, the aggregated cost *C_A_* is obtained by

(7) $$C_{A}(p,d)=\frac{\sum_{q\in N(p)}\delta (p,q)\times w_{s}(p,q)\times C(q,d)}{\sum_{q\in N(p)}\delta (p,q)\times w_{s}(p,q)},$$

where *δ*(·) represents an indicate function which identifies whether **p** and **q** belong to the same segment or not as follows:

(8) $$\delta (p,q)=\{\begin{array}{cc} 1, & \mathrm{if}\;G_{a}(p)=G_{a}(q)\\ 0, & \mathrm{otherwise} \end{array},$$

where *G_a_* represents the segmented AV image [26]. In addition, to reduce the computational complexity of the cost aggregation step, cost aggregation is performed for the selected disparity candidates [27].

Finally, the WTA optimization is performed to obtain the best disparity as given below:

(9) $$D(p)=\underset{d}{\arg\max}C_{A}(p,d).$$

### 2.3. Hole-Filling for the Warped AV HDR Image

**Figure 8:**
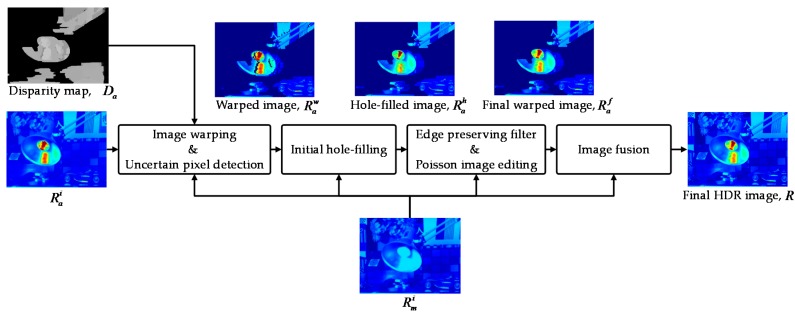
A flow chart of the hole-filling and the fusion process.

**Figure 9:**
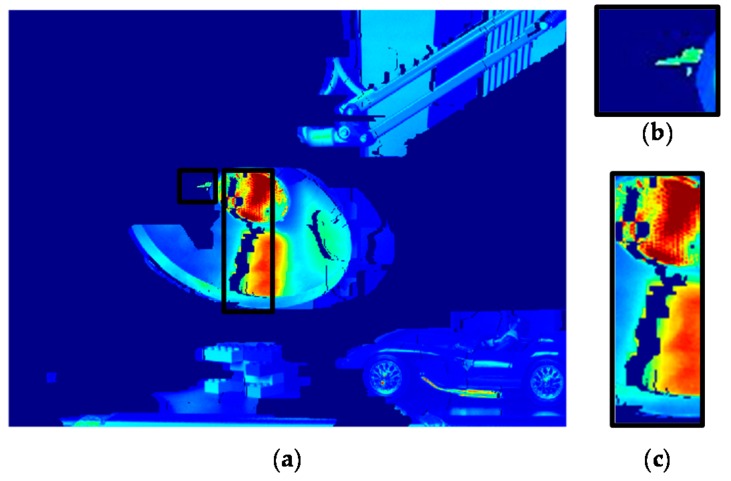
(**a**) $R_{a}^{w}$; (**b**) example of incorrectly warped part enlarged with a black box in (**a**); and (**c**) example of holes enlarged with black box in (**a**).

Figure 8 presents a flow chart of the warping, hole-filling, and image fusion process for reconstructing the final HDR image. Given the AV disparity *D_a_*, the interest pixels in the AV HDR image can be aligned with the MV HDR. To do this, forward warping is performed using *D_a_*, as follows:

(10) $$R_{a}^{w}\left(p^{\prime }\right)=R_{a}^{i}(p)$$

where **p** = [*x y*]^T^ and **p′** = [(*x* + *D_a_*(**p**)) *y*]^T^, and $R_{a}^{i}$ and $R_{a}^{w}$ denote the initial AV HDR image and the warped AV HDR image, respectively. However, due to inaccurate disparity values and occlusion, $R_{a}^{w}$ involves incorrect radiance values and holes as shown in Figure 9b,c. Thus, uncertain pixels with the incorrect radiance value should be detected and removed. Then, the radiance values of holes can be restored using reliable radiance values.

**Figure 10:**
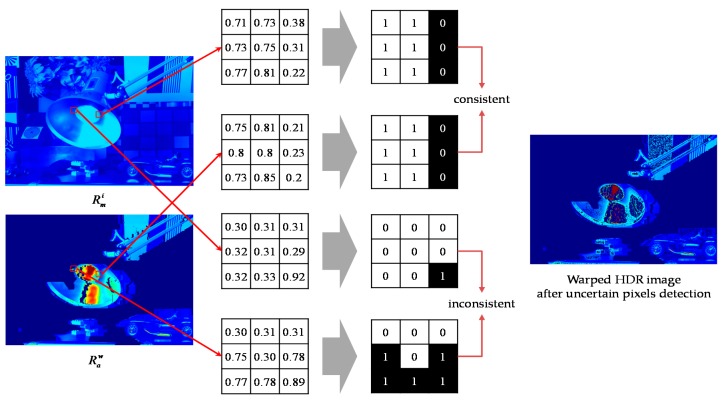
An example of the uncertain pixel detection.

To detect unreliable pixels, the structure of each pixel in $R_{a}^{w}$ is compared with that of co-located pixel in the initial MV HDR image $R_{m}^{i}$ using the structural similarity proposed in [28]. For each HDR image, the nine pixels inside a 3 × 3 patch centered on each pixel are converted into a bit-string by thresholding with the average of the radiance values inside the patch. The radiance value of the pixel is preserved if two bit-strings obtained from the co-located pixel in $R_{m}^{i}$ and $R_{a}^{w}$ are equal. Otherwise, the pixel radiance value is replaced with zero, as shown in Figure 10. In other words, the detected uncertain pixels are turned into the holes.

**Figure 11:**
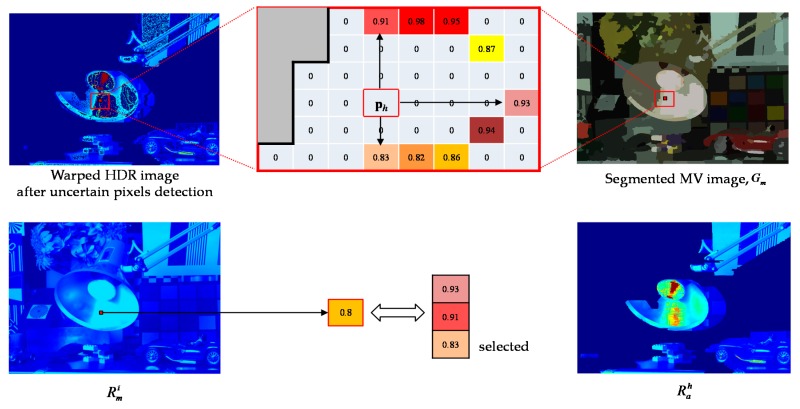
An example of initial hole-filling.

Before proceeding to the image fusion process, it is required to restore the radiance value of the hole. To achieve this purpose, an effective hole-filling method is proposed. Let Ω represent a set of holes. To determine the restored radiance value of the pixel in hole **p***_h_* ∈ Ω, the rays along the four directions from **p***_h_* are first emitted. When each ray from **p***_h_* meets the pixel **q** ∉ Ω, the radiance value of **q** is collected as the candidate radiance value of **p***_h_*. Moreover, to collect only reliable candidate pixels, the segmented image is used. It can be assumed that the pixels in the same segment have the similar radiance values. Thus, the candidate pixel **q** is only collected inside the same segment which includes **p***_h_*. For example, as shown in Figure 11, **p***_h_* has only three candidate radiance values. The radiance value of **p***_h_* is determined as one among the candidate values that is most similar to the radiance value of the co-located pixel in $R_{m}^{i}$.

**Figure 12:**
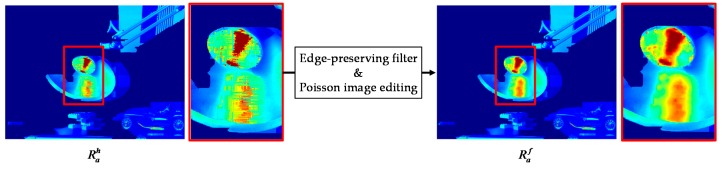
Restoration radiance values for holes.

Next, a filtering process is performed to improve the overall radiance. The edge-preserving filter [29] is applied for smoothing the hole-filled HDR image, $R_{a}^{h}$, by using the gradient of $R_{m}^{i}$ as guidance. To further recover the texture information lost by the filtering process, Poisson image editing [30] is used as a secondary post-processing. Specifically, Poisson image editing is utilized to transfer the gradients of $R_{m}^{i}$ to the gradients in Ω. To this end, the solution of the minimization problem is defined as follows:

(11) $$R_{a}^{f}=\underset{R_{a}^{e}}{\min}{\iint_{\Omega }\left|\nabla R_{a}^{e}-\nabla R_{m}^{i}\right|}^{2}\;\mathrm{with}\;{\;R_{a}^{e}|}_{\partial \Omega }={R_{a}^{f}|}_{\partial \Omega },$$

where $R_{a}^{e}$ represents the resultant HDR image of the edge-preserving filtering process [29], ∇ denotes the gradient operator, and *∂*Ω represents the boundary of Ω. The final warped HDR image $R_{a}^{f}$ is shown in Figure 12.

**Figure 13:**
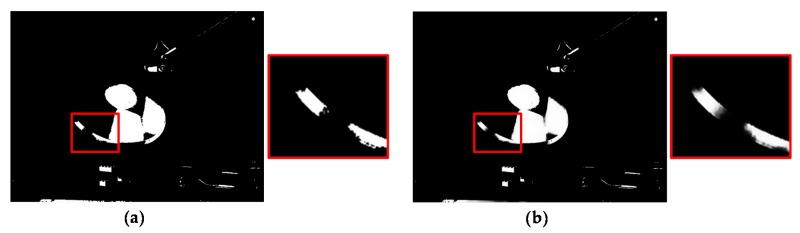
(**a**) Under/over-exposed region map, *S*; and (**b**) weight map, *W*.

Finally, given $R_{a}^{f}$, the image fusion process is performed so that the radiance values of the under/over-exposed regions are determined by those of $R_{a}^{f}$ and the radiance values of the rest regions are obtained from those of $R_{m}^{i}$. That is, the final HDR image *R* is reconstructed by fusing $R_{m}^{i}$ with $R_{a}^{f}$, as follows:

(12) $$R\left(p\right)=\left(1-W(p)\right)\times R_{m}^{i}(p)+W(p)\times R_{a}^{f}(p),$$

where *W*(·) is the weight map for image fusion. To blend the two HDR images seamlessly around the boundary of the under/over-exposed region, the weight map *W* is defined by smoothing the under/over-exposed region map *S* using the edge-preserving filter [29], which preserves strong edges of $R_{m}^{i}$, as shown in Figure 13.

## 3. Results

### 3.1. Experimental Setup

To evaluate the performance of the stereo HDR imaging, the experiments were conducted on four stereo datasets: three datasets (*Aloe*, *Art*, and *Moebius*) of the Middlebury database [31] and the *IIS Jumble* dataset used in [16]. Each dataset of the Middlebury database consists of seven views (View 0–6) with three different exposure times for three illumination types (Illum 1–3). For each dataset, a chosen illumination was used for the experiments. The *Aloe* dataset has a resolution of 641 × 555 with Illum 3. The *Art* and *Moebius* datasets have a resolution 695 × 555 with Illum 2 and Illum 1, respectively. Among the seven views in the Middlebury database, View 1 and View 5 were chosen as the left-view and right-view, respectively, for the stereo HDR imaging. The *IIS Jumble* dataset is comprised of 15 different views with a resolution of 2560 × 1920. On the IIS Jumble dataset, the images from View 12 and View 13 were chosen, down-sampled by a factor of 2 in each dimension, and cropped to 800 × 600. All the experiments were conducted with the left-view images as MV images and right-view images as AV images.

All the parameters used in the proposed method were experimentally determined. To detect the under/over-exposed regions, *τ_u_* and *τ_o_* in Equation (1) were set to 5 and 250. For SL segmentation [20], the strip size and energy tolerance were set to 6 and 4. Furthermore, in the GRM method [21], filter variation, control value, and the minimum size of the segment were set to 0.1, 150, and 1, respectively. In the disparity estimation process, the window size for the census transform-based cost computation was set to 7 and the disparity search ranges, *d*_max_, of the *Aloe*, *Art*, *Moebius*, and *IIS Jumble* were set to 100, 120, 120, and 100 pixels, respectively. The number of disparity candidates was set to 10 percent of *d*_max_ and *σ* was set to 17 in Equation (6). The spatial and range standard deviations for the edge-preserving filter [29] were, respectively, set to 20 and 0.0005, and the number of iteration steps was set to 2.

### 3.2. Evaluation of Performance

**Figure 14:**
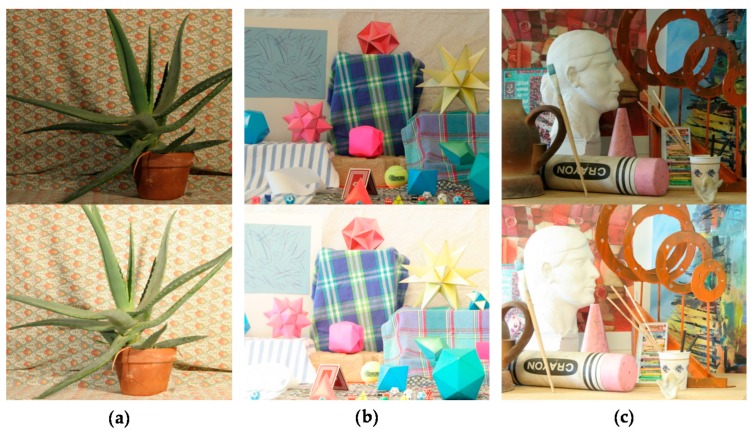
Input image pairs with normal-long exposure from the Middlebury database: (**a**) *Aloe*; (**b**) *Moebius*; and (**c**) *Art*.

**Figure 15:**
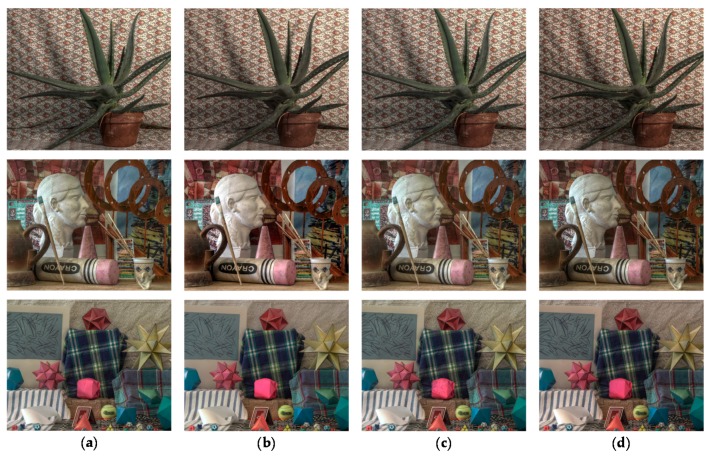
Resultant LDR images for the Middlebury database with normal-long exposure: (**a**) reference image; (**b**) Lin and coworkers’ method [14]; (**c**) Batz and coworkers’ method [16]; and (**d**) proposed method.

**Figure 16:**
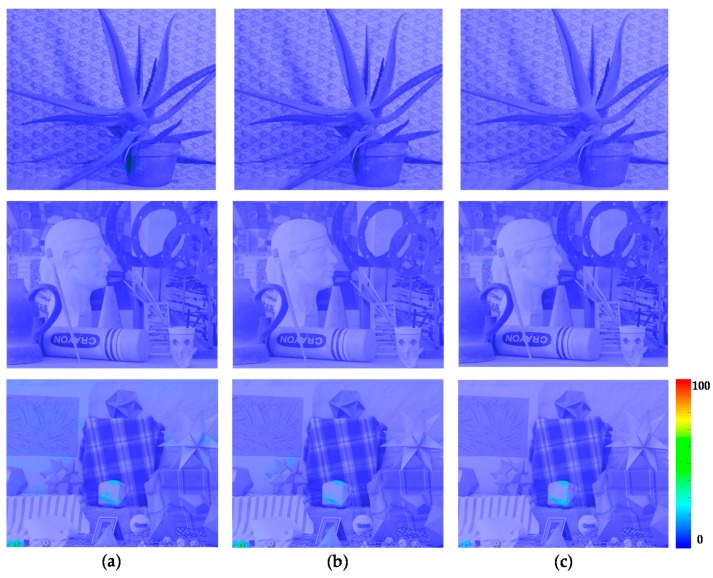
Resultant HDR visual difference predictor 2 (HDR-VDP2) maps for the Middlebury database with normal-long exposure: (**a**) Lin and coworkers’ method [14]; (**b**) Batz and coworkers’ method [16]; and (**c**) proposed method.

In order to compare the performance of the proposed stereo HDR imaging to the conventional method, the experiments were performed in two different exposure settings, the normal-long exposure and the short-long exposure. In the case of the normal-long exposure, the images with the normal exposure time and the long exposure times were used as input images, as shown in Figure 14. In the *Aloe* dataset, the exposure times of the input images are 500 ms and 2000 ms, respectively. In the *Art* and *Moebius*, the exposure times of those are 1000 ms and 4000 ms. Figure 15 shows the reference tone-mapped LDR images and the resultant tone-mapped LDR images obtained by the conventional method and the proposed method. In this paper, all the resulting HDR images were tone-mapped for visualization on the LDR display devices using the tone mapping operator [32], which was also used in the conventional method [16]. As shown in Figure 15, the superiority of the proposed method over the conventional methods [14,16] is not very convincing from the tone-mapped versions of the HDR images in the case of the normal-long exposure. To compare the objective quality of the resultant HDR images, the visual difference predictor (VDP), called HDR-VDP-2, which is a well-known image quality metric for HDR image [33], was employed. The HDR-VDP-2 ranges from 0 (worst) to 100 (best) The reference HDR images were generated using the HDR imaging method based on the bracketed images [16,34]. Table 1 shows the obtained HDR-VDP-2 scores. The proposed method achieved 2.0 and 1.3 more points than the methods of Lin et al. [14] and of Batz et al. [16] in the case of normal-long exposure, respectively. In addition, the HDR-VDP2 maps were presented for better visualization. The HDR-VDP2 maps are color-coded using the color range which represents the error values (from 0 to 100). As shown in the color bar of Figure 16, the blue and red colors represent the lowest (0) and highest (100) error values, respectively. Figure 16 shows that the differences among the conventional methods [14,16] and the proposed algorithm are marginal in the normal-long exposure case.

**Figure 17:**
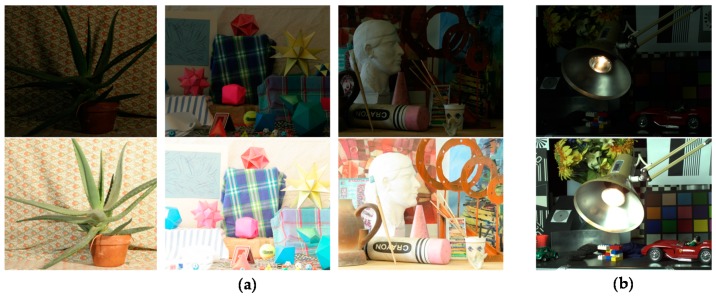
Input image pairs with short-long exposure: (**a**) input Middlebury images; and (**b**) input *IIS Jumble* images.

**Figure 18:**
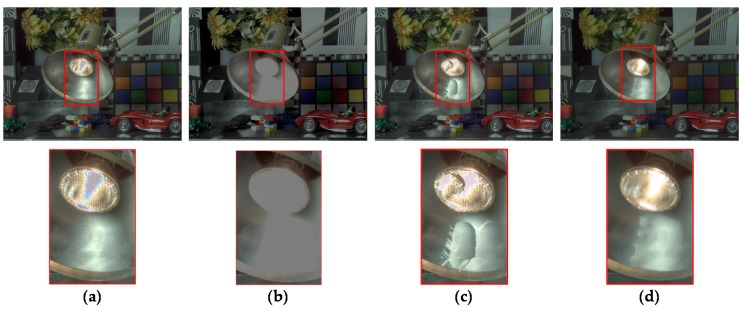
Resultant LDR images for the *IIS Jumble* image: (**a**) reference image; (**b**) Lin and coworkers’ method [14]; (**c**) Batz and coworkers’ method [16]; and (**d**) proposed method.

**Figure 19:**
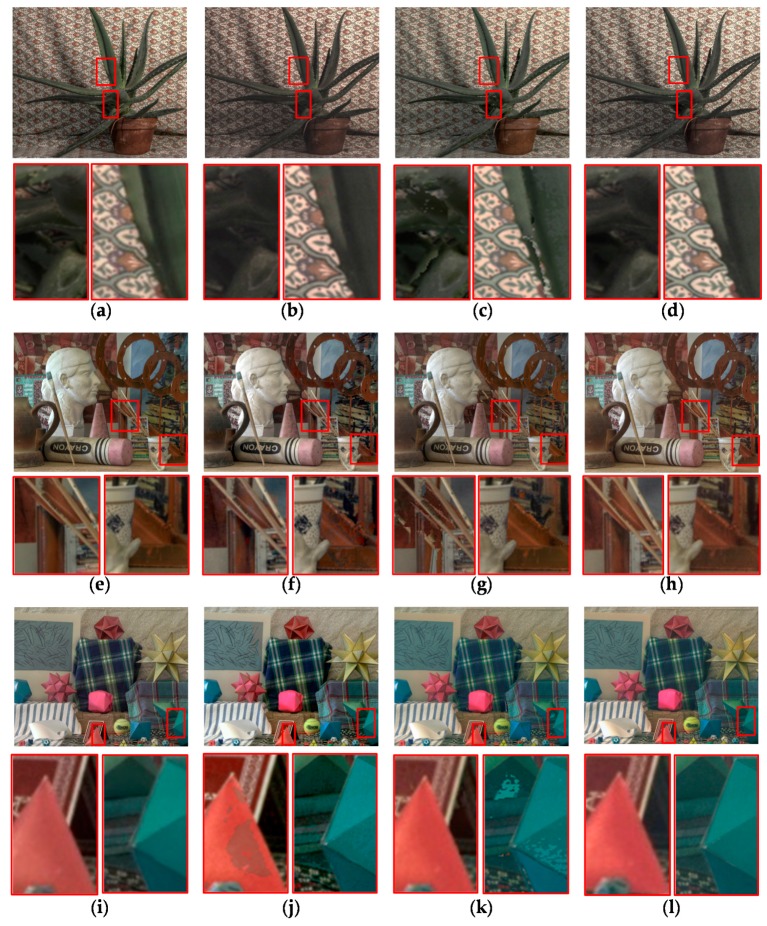
Resultant LDR images for the Middlebury database with short-long exposure: (**a**,**e**,**i**) reference image; (**b**,**f**,**j**) Lin and coworkers’ method [14]; (**c**,**g**,**k**) Batz and coworkers’ method [16]; and (**d**,**h**,**l**) proposed method.

**Figure 20:**
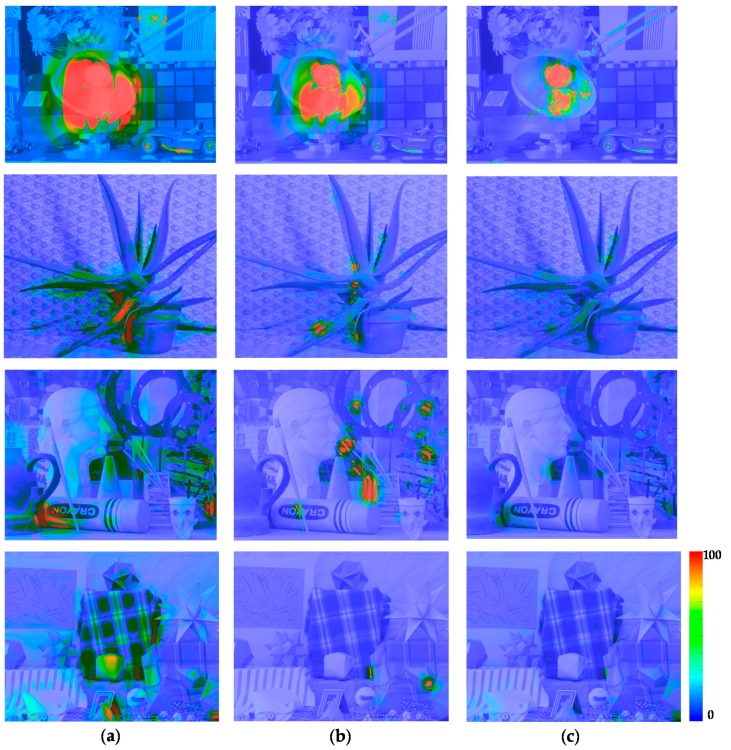
Resultant HDR-VDP2 maps for the Middlebury database and the *IIS Jumble* dataset with short-long exposure: (**a**) Lin and coworkers’ method [14]; (**b**) Batz and coworkers’ method [16]; and (**c**) proposed method.

In Figure 17, the first row and the second row represent the MV and the AV images captured by short and long exposure times, respectively. In the *Aloe* dataset, the exposure times of the input images are 125 ms and 2000 ms. In the *Art* and *Moebius*, the exposure times are 250 ms and 4000 ms, respectively. The exposure times of the *IIS Jumble* are 61 ms and 5 ms. Figure 18 shows the resultant LDR images of the *IIS Jumble* dataset in the case of short-long exposure. To highlight the differences, certain parts of the resultant images were indicated by red rectangles. As shown in Figure 18b, the Lin and coworkers’ method [14] failed to reconstruct the radiance values in the over-exposed region around the light bulb. The Batz and coworkers’ method [16] reconstructed the resultant HDR image with clearly visible artifacts in those regions, as shown in Figure 18c. On the other hand, the proposed method provided the reconstructed image without obvious artifacts as show in Figure 18d. Figure 19 shows the results of the conventional methods and the proposed method for the Middlebury database. In the *Aloe* dataset, as shown in Figure 19d, it seems that the Lin and coworkers’ method [14] generated the HDR images without artifacts, but the method could not sufficiently restore the radiance values, as listed Table 1. The Batz and coworkers’ method [16] generated artifacts at object boundaries, as shown in the Figure 19g. On the other hand, the proposed method generated the resultant image with clear object boundaries, as shown in the Figure 19j. The similar artifacts could be observed in the result of the *Art* dataset, as shown in Figure 19f,g. Lin and coworkers’ method [14] made the artifacts in the right-bottom regions magnified by red rectangle. In the resultant image of Batz and coworkers’ method [16], the parts of sticks of brushes and red pillars appeared repeatedly. In contrast, the proposed method reconstructed the resultant image without such artifacts, as shown in Figure 19k. In the *Moebius* dataset, while the conventional methods generated the artifacts such as those in the magnified regions, the proposed method reconstructed the HDR image without obvious artifacts at the object boundaries, as shown in last row of Figure 19. The objective quality of the resultant HDR images of the proposed method and the conventional algorithms [14,16] in the Middlebury database and the *IIS Jumbl*e dataset are listed in Table 1. On average, the proposed method achieved a gain of 13.6 and 8.3 points in the HDR-VDP-2 quality score as compared to the methods of Lin et al. [14] and of Batz et al. [16], respectively. Figure 20 shows the HDR-VDP2 maps of the resultant images of the Middlebury database and the *IIS Jumble* dataset in the case of short-long exposure. The superiority of the proposed method over the conventional methods is clearly noticeable in the short-long exposure case as shown in Figure 20. For example, the HDR-VDP-2 maps of the *IIS Jumble* dataset obtained by the conventional methods exhibit large values around the over-exposed regions near the light bulb.

**Figure 21:**
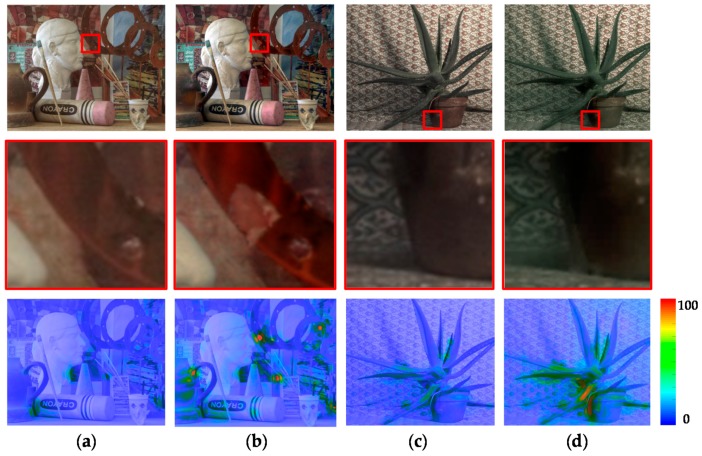
Resultant LDR images (first row) and their magnified sub-regions (second row) obtained with the two different ICRF estimation methods: (**a**,**c**) proposed ICRF estimation; and (**b**,**d**) conventional ICRF estimation [14]. Color-coded HDR-VDP2 maps (last row) are provided for a better performance comparison.

**Figure 22:**
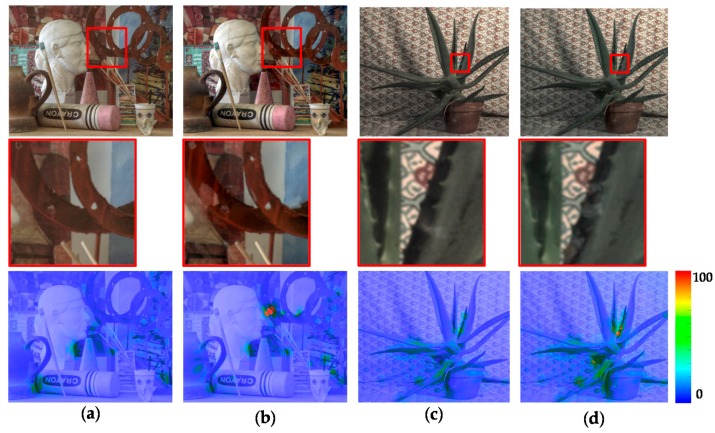
Resultant LDR images (first row) and their magnified sub-regions (second row) obtained with or without the proposed rejection step: (**a**,**c**) with the proposed rejection step; and (**b**,**d**) without the proposed rejection method. Color-coded HDR-VDP2 maps (last row) are provided for a better performance comparison.

**Figure 23:**
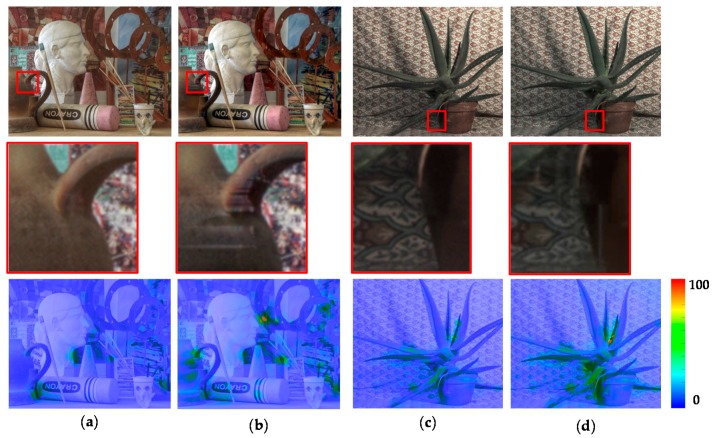
Resultant LDR images (first row) and their magnified sub-regions (second row) obtained with or without Poisson image editing (PIE): (**a**,**c**) with PIE; and (**b**,**d**) without PIE. Color-coded HDR-VDP2 maps (last row) are provided for a better performance comparison.

In addition, we performed an in-depth analysis of the components of the proposed method including the ICRF estimation, rejection of the uncertain pixels, and Poisson image editing. To this end, the experiments were conducted when each method is excluded or replaced by other conventional method. Figure 21 shows the resultant images and the HDR-VDP2 maps obtained using the proposed and conventional ICRF estimation methods. The conventional method [14] generates the HDR images with clearly visible artifacts especially around object boundaries, as shown in the second row of Figure 21. The HDR-VDP2 maps clearly show the strength of the proposed method over the conventional method. Figure 22 shows that the rejection of the uncertain pixels can reduce the artifacts resulting from inaccurate disparity values. Figure 23 shows the effects of Poisson image editing. In the proposed method, Poisson image editing step serves to further remove artifacts, as shown in Figure 23. For the quantitative performance evaluation, we measured the HDR-VDP2 scores for the sub-optimal configurations, as listed in Table 2. For notational simplicity, the rejection of the uncertain pixels and Poisson image editing are represented as rejection and PIE, respectively. It can be seen that each method is essential for reconstructing a high quality HDR image and the proposed method that includes all the methods yields the best performance.

## 4. Conclusions

In this paper, the method to reconstruct an HDR image was presented using stereo LDR images with different exposure times. Since the HDR image is reconstructed from images simultaneously captured with different exposure, the stereo HDR imaging method has a merit of being relatively free from the ghosting artifact problem in comparison with the HDR imaging method based on the temporal exposure bracketing. However, the performance of the stereo HDR imaging depends on the following processes: ICRF estimation to obtain the initial HDR images, disparity estimation to align the input images, and image warping followed by image fusion to reconstruct a high-quality HDR image. Unlike the conventional stereo HDR imaging methods, the proposed method mainly improved two major sub-processes: (1) disparity estimation; and (2) image warping followed by image fusion. In the disparity estimation process, the disparity was estimated only at the pixels in the interest regions detected using the segmented image. Moreover, the HVS-based cost computation and segmentation-based cost aggregation were proposed to accurately estimate the disparity. In the image warping and fusion process, effective hole-filling was performed to enhance the warped HDR image. Then, the final HDR image was reconstructed through edge-preserving filter-based image fusion. The experimental results demonstrated the superiority of the proposed stereo HDR imaging method compared to the conventional method.

## Figures and Tables

**Table 1:** Quantitative performance evaluation from the HDR-VDP-2 quality score [32].

HDR-VDP-2	Normal-Long Exposure Pairs			Short-Long Exposure Pairs		
	Lin and Coworkers’ Method [14]	Batz and Coworkers’ Method [16]	Proposed	Lin and Coworkers’ Method [14]	Batz and Coworkers’ Method [16]	Proposed
*Art*	92.05	92.76	94.87	79.23	84.95	92.64
*Aloe*	91.51	92.81	94.30	78.89	83.81	90.57
*Moebius*	92.27	92.44	92.76	82.43	86.26	92.71
*IIS Jumble*				38.81	45.56	58.01
**Average**	91.94	92.67	**93.98**	69.84	75.15	**83.48**

**Table 2:** In-depth quantitative performance evaluation of the proposed method with different configurations.

HDR-VDP-2	*Art*	*Aloe*	*Moebius*	*IIS Jumble*
*Proposed*	92.64	90.57	92.71	58.01
*Proposed with conventional ICRF*	86.61	81.69	87.25	52.95
*Proposed without Rejection*	84.91	86.25	84.56	41.93
*Proposed without PIE*	86.44	86.66	87.78	49.57
*Proposed without Rejection & PIE*	82.48	85.81	82.63	36.22
*Proposed with conventional ICRF & without PIE*	82.26	78.18	85.90	49.88
*Proposed with conventional ICRF & without Rejection*	81.34	78.35	84.68	38.84
*Proposed with conventional ICRF & without Rejection and PIE*	78.17	77.95	81.87	35.96
